# Complement deposition, C4d, on platelets is associated with vascular events in systemic lupus erythematosus

**DOI:** 10.1093/rheumatology/keaa092

**Published:** 2020-04-07

**Authors:** Elisabet Svenungsson, Johanna T Gustafsson, Giorgia Grosso, Marios Rossides, Iva Gunnarsson, Kerstin Jensen-Urstad, Anders Larsson, Kristina N Ekdahl, Bo Nilsson, Anders A Bengtsson, Christian Lood

**Affiliations:** k1 Division of Rheumatology, Department of Medicine Solna, Karolinska Institutet, Karolinska University Hospital, Stockholm; k2 Division of Clinical Epidemiology, Department of Medicine Solna, Karolinska Institutet, Stockholm; k3 Department of Clinical Physiology, Karolinska Institutet, Södersjukhuset, Stockholm; k4 Department of Medical Sciences, Clinical Chemistry, Uppsala University, Uppsala; k5 Department of Immunology, Genetics and Pathology, Uppsala University, Uppsala; k6 Linnaeus Center of Biomaterials Chemistry, Linnaeus University, Kalmar; k7 Department of Clinical Sciences Lund, Section of Rheumatology, Lund University, Lund, Sweden; k8 Department of Medicine, Division of Rheumatology, University of Washington, Seattle, WA, USA

**Keywords:** systemic lupus erythematosus, antiphospholipid syndrome, C4d, vascular events, antibodies, risk factors

## Abstract

**Objective:**

Complement components, including C4d, can be found on activated platelets, a process associated with vascular disease in SLE. We investigated whether platelet C4d (PC4d) adds additional value to traditional and known lupus-associated risk factors when identifying SLE patients with vascular disease.

**Methods:**

This cross-sectional study included 308 well-characterized SLE patients and 308 matched general population controls. PC4d deposition was analysed using flow cytometry. Values >95% of controls were considered as PC4d positive (+). aPL were determined by Luminex, and the LA test was performed by DRVVT. History of vascular disease (composite and as separate outcomes) was defined at inclusion.

**Results:**

SLE patients had increased PC4d deposition as compared with population controls (50 *vs* 5%, *P* < 0.0001). PC4d+ positively associated with any vascular events, and separately with venous and cerebrovascular events, and also with all investigated aPL profiles. The association for any vascular event remained statistically significant after adjustment for traditional and SLE-associated risk factors (odds ratio: 2.3, 95% CI: 1.3, 4.3, *P* = 0.008). Compared with patients negative for both PC4d and LA, patients with double positivity were more likely to have vascular disease (odds ratio: 12.3, 95% CI: 5.4, 29.3; attributable proportion due to interaction 0.8, 95% CI: 0.4, 1.1)

**Conclusion:**

PC4d+ is associated with vascular events in SLE, independently of traditional and SLE-associated risk factors. Concurrent presence of PC4d and LA seem to interact to further increase the odds for vascular events. Prospective studies should examine whether the aPL/PC4d combination can improve prediction of vascular events in SLE and/or APS.

**Rheumatology key messages**

- SLE patients have elevated deposition of C4d on platelets as compared with controls.
- In SLE patients, platelet C4d is associated with vascular events, independent of traditional risk factors.
- Platelet C4d and LA seems to interact synergistically to increase the likelihood of vascular events.

## Introduction

The classical pathway of the complement system is important for a non-inflammatory clearance of apoptotic cells and immune complexes [1], limiting the production of pro-inflammatory cytokines, including type I IFNs [2]. However, the complement system also generates activation split products, which mediate chemotaxis and damage through initiation of an inflammatory state. Complement is furthermore an important regulator of the coagulation system, providing an essential link to thrombotic disease [3, 4]. Activity in the complement system can thus cause both inflammation and thrombosis.

We, and others, have demonstrated that genetic deficiency of the early components of the classical complement pathway, including C1q and C4 [5], is strongly associated with the development of SLE [6], an autoimmune rheumatic disease with a marked increased risk of vascular morbidity and mortality, which is not fully explained by traditional risk factors. The relative cardiovascular risk is known to be especially high among young women with lupus [7]. Venous thromboembolism is also common in SLE, especially during the first years after diagnosis [8]. The APS, an acquired thrombophilia, is observed in 10–20% of SLE patients. It is defined by the simultaneous occurrence of aPL, present in 30–50% of SLE patients, together with vascular events and/or pregnancy morbidity [9]. Several prospective studies have demonstrated that the occurrence of aPL is a risk factor for vascular events in SLE [10–12].

There is now mounting evidence that complement activation is important for the development of thrombosis and pregnancy morbidities in APS [13–15]. Several studies have implicated that complement plays a role for the extent of tissue damage after vascular events also in the general population [16]. When aPL bind to platelets they induce platelet activation and simultaneously provide complement-fixing antibodies on the platelet surface [17]. Upon platelet activation, frequently observed in SLE patients [18–20], platelets expose surfaces allowing for complement activation [21, 22]. Once activated, the complement cascade will produce pro-inflammatory split products, C3a and C5a, contributing to chemotaxis and local inflammation, furthering platelet, monocyte and endothelial activation, all contributing to a procoagulant state [23, 24]. We, and others, have demonstrated that platelets from SLE patients are highly opsonized with complement components, including C1q, C4d and C3d; the latter two are degradation products of C4b and C3b [17, 25, 26]. Deposition of C4d on platelets (PC4d) seems to be fairly specific for SLE [26], though it is also reported in non-lupus individuals with stroke [27], as well as in other rheumatic diseases, including RA and SSc [17]. Enhanced PC4d depositions have been associated with thrombosis, both arterial and venous, and they are suggested to have prognostic value [17, 25–29]. Though clearly associated with vascular disease, it is not known whether PC4d adds clinical value to the current panel of markers, e.g. traditional cardiovascular risk factors and the more lupus-specific risk factors such as aPL and nephritis/impaired renal function, when assessing thrombotic risk in SLE.

In this cross-sectional study, we evaluated C4d on platelets from >300 SLE patients and matched general population controls. We studied the performance of PC4d as a risk factor for vascular disease, adjusting for traditional and lupus-associated risk factors. Our results demonstrate that PC4d is an independent marker of vascular events in SLE, which alone, and combined with positivity in the functional LA test, adds significant clinical value for assessing vascular events in these patients.

## Methods

### Patients

Consecutive patients with SLE (*n* = 308) from the Karolinska lupus cohort and general population controls (*n* = 308), matched for age, sex and region of living to the SLE patients, were recruited to participate in studies related to cardiovascular disease. All SLE patients fulfilled at least four of the revised ACR 1982 criteria for SLE [30]. Patients and controls were investigated in person by a rheumatologist and a nurse according to similar structured protocols. Overviews of the clinical characteristics of the patients have been reported previously [19, 31, 32]. Treatment was recorded at inclusion. Disease activity was assessed using SLAM [33] and SLEDAI-2000 (SLEDAI-2K) [34]. Organ damage was determined by the Lupus International Collaborative Clinics/ACR Damage Index [35]. Vascular disease at inclusion was objectively verified and defined as previously described [32], and discussed [36]. Definitions are also detailed in Supplementary Material, section Definition of vascular events, available at *Rheumatology* online. Ischaemic heart disease (IHD, including myocardial infarction and angina), ischaemic cerebrovascular disease (ICVD, including ischaemic stroke and transitory ischaemic attacks), venous thromboembolism (VTE, including deep venous thrombosis and pulmonary embolism) were considered separately as well as a composite outcome (any vascular event). Carotid plaques occurrence was used as a measure of atherosclerosis. Traditional cardiovascular risk factors incluging age, gender, smoking, hypertension (systolic blood pressure ≥140 or diastolic pressure ≥90 mmHg, or antihypertensive treatment due to high blood pressure) were assessed at the inclusion visit to the cohort. The study complies with the Declaration of Helsinki and it was approved by the Local Ethics Committee of the Karolinska University Hospital/Karolinska Institutet, Stockholm, Sweden (number 03-556). All participants gave informed written consent to participate.

### Clinical chemistry

Routine clinical chemistry was performed by the laboratory at Karolinska University Hospital.

Cystatin C was analysed on an Architect Ci8200 analyzer (Abbott Laboratories, Abbott Park, II, USA), with reagents from Gentian (Moss, Norway). Vascular cell adhesion molecule 1 (VCAM-1; DY809), Interferon (IFN)-γ inducible protein (IP-10, DY266) and IL-6 (DY206) were analysed with commercial sandwich ELISA kits (R&D Systems, Minneapolis, MN, USA).

Complement factors C3, C4 and C3dg were analysed in EDTA-plasma on a Modular analyzer (Roche). Complement soluble (s)C5b-9 was measured by in house ELISA using monoclonal anti-Hu-C9 aEII (Bioporto Diagnostics A/S, Hellerup, Denmark) for capture and polyclonal biotinylated anti-Hu-C5 (sheep) antibody BP373 (Acris, Herford, Germany) for detection [37]. Pooled human serum was incubated with washed zymosan 1 mg/mL (Sigma, St Louis, MO, US) at 37°C for 30 min. Thereafter complement activation was stopped by the addition of 10 mM EDTA. The level of sC5b-9 were calibrated using a commercially available EIA kit (Microvue, Quidel, San Diego, CA, US).

### Autoantibodies

Autoantibodies targeting phospholipids/cofactors (aCL and β_2_glycoprotein-I of IgG, IgM, IgA isotypes) and specific nuclear antigens (dsDNA, Smith, RNP 68, SSA/Ro52, SSA/Ro60, SSB) were analysed by multiplexed bead technology (Luminex) using the BioPlex 2200 system (Bio-Rad, Hercules, CA, USA) according to the specifications of the manufacturer. The cut-off for aCL and anti-β_2_glycoprotein-I was set at the 99th percentile of the normal population, according to APS criteria [9]. The coefficient of variation % was <8.0 Units/ml for all aPL isotypes. For the specific anti-nuclear antigens, we used cut-offs for positivity as specified by the manufacturer. LA was determined using a modified DRVVT (Biopool) using Bioclot LA. In addition, plasma was collected and stored at –70°C in a central biobank at Karolinska Institutet.

### Complement deposition on platelets

Platelets were isolated as described previously [17, 19, 20]. Briefly, platelet-rich plasma was obtained through centrifugation of anticoagulated (sodium citrate) blood at 280*g* for 10 min. The platelet-rich plasma was stored at –80°C until used. Complement deposition on platelets was not affected by freeze–thawing, with similar PC4d levels observed on freshly isolated platelets as compared to frozen platelets (*r* = 0.88, *P* = 0.003, supplementary Fig. S1, available at *Rheumatology* online), consistent with what has been described for other platelet proteins previously [19]. The platelet-rich plasma was mixed with 10 mM EDTA in PBS and centrifuged at 1125*g* for 10 min to pellet the platelets. For detection of C4d fragments, platelets were stained with anti-C4d antibodies (Quidel, San Diego, CA, USA) followed by a FITC-conjugated rabbit-anti-mouse IgG antibody (Dako, Glostrup, Denmark). Platelets were identified by staining with anti-CD42a antibodies, and analysed by flow cytometry on an Accuri C6 (BD). The cut-off for high C4d deposition was determined by the 95th percentile of the population controls. Platelet CD69 expression was analysed as described previously [18].

### Carotid ultrasound

Atherosclerosis was evaluated with carotid US using a duplex scanner with a linear array transducer, as previously described [32]. Both right and left carotids were measured and presence of plaque was defined as ≥100% increase over background in any arterial segment.

### Statistics

Patient/control characteristics are presented as median (interquartile range) or as percentages.

For comparisons between groups, the χ^2^ or the Mann–Whitney *U* test were used as appropriate. We used logistic regression models reporting odds ratios (ORs) and corresponding 95% CIs to estimate associations between investigated variables and PC4d+, LA+ and vascular events (separately and as composite outcomes). We also tried a stricter cut-off (99th percentile), yielding very similar results.

To determine whether PC4d was independently associated with vascular disease, we analysed models adjusted for traditional cardiovascular risk factors (age, gender, hypertension and smoking). To avoid multicollinearity among the lupus-related risk factors, we grouped variables describing similar functions and chose the variable with the lowest *P*-value to include in the multivariable model (supplementary Table S1, available at *Rheumatology* online). Thus, we added LA and glomerular filtration rate according to the Modification of Diet in Renal Disease as proxies for aPL and renal involvement, respectively. We also added steroid treatment. The number of outcome events limited the number of variables that we could adjust for in the models.

Last, we examined possible additive interaction between PC4d and LA on the odds of vascular disease, after adjusting for traditional risk factors and steroid treatment, by estimating the relative excess risk due to interaction and the attributable proportion due to interaction [38]. Data were analysed using JMP version 14.2.0 (SAS Institute Inc., Cary, NC, USA) and R version 3.6.1 (R Foundation for Statistical Computing, Vienna, Austria). A *P*-value <0.05 was considered statistically significant.

## Results

### Characteristics of patients and controls

SLE patients and controls were well matched for age and gender. Vascular events, both arterial and venous, were more common among patients than controls. Demographics, clinical and laboratory characteristics for SLE patients and controls are presented in Table 1.

**Table 1:** Baseline demographics, complement, autoantibodies and inflammatory biomarkers in patients and control subjects

	SLE patients (*N* = 308), *N* (%) or median (IQR)	Controls (*N* = 308), *N* (%) or median (IQR)	*P*-value
Demographics and traditional risk factors			
Age (years)	48 (35–58)	48 (35–58)	0.82
Female sex	284 (92)	283 (92)	0.99
Current smoking	57 (19)	46 (15)	0.23
Hypertension	134 (44)	61 (20)	**<0.0001**
Vascular events			
Any vascular event (arterial or venous)	84 (27)	11 (4)	**<0.0001**
Any arterial event	48 (16)	6 (2)	**<0.0001**
Ischemic heart disease	21 (7)	1 (0.3)	**<0.0001**
Myocardial infarction	15 (5)	0 (0)	**<0.0001**
Ischaemic cerebrovascular disease	30 (10)	3 (1)	**<0.0001**
Ischaemic stroke	24 (8)	3 (1)	**<0.0001**
Venous thromboembolism	45 (15)	5 (2)	**<0.0001**
Carotid plaques	57 (20)	46 (17)	0.26
Lupus manifestations and characteristics			
Disease duration (years)	12.9 (6.0–22.6)	–	ND
Malar rash	167 (54)	–	ND
Photosensitivity	212 (69)	–	ND
Discoid lesions	60 (20)	–	ND
Oral ulcers	108 (35)	–	ND
Arthritis	257 (83)	–	ND
Serositis	120 (39)	–	ND
Nephritis	127 (41)	–	ND
CNS manifestation	36 (12)	–	ND
Leucopenia	155 (51)	–	ND
Thrombocytopenia	64 (21)	–	ND
SDI damage index >1	197 (64)	–	ND
SLAM >6	186 (60)	–	ND
SLEDAI >6	100 (32)	–	ND
Treatment (at inclusion)			
Steroids	173 (56)		
Antimalarial agents	99 (32)		
aPL/disorders (positivity)			
aCL IgG	73 (24.7)	2 (0.7)	**<0.0001**
aCL IgM	16 (5)	1 (0.3)	**<0.0001**
aCL IgA	50 (16)	1 (0.3)	**<0.0001**
aβ_2_GPI IgG	80 (26)	2 (0.7)	**<0.0001**
aβ_2_GPI IgM	22 (7.1)	1 (0.3)	**<0.0001**
aβ_2_GPI IgA	48 (16)	1 (0.3)	**<0.0001**
LA	50 (16)	–	ND
Any aPL	96 (31)	–	ND
Persistent aPL	66 (22)	–	ND
Triple aPL positivity	42 (14)	–	ND
APS	29 (10)	–	ND
Other autoantibodies (positivity)			
ANA (ever)	303 (99)	–	ND
dsDNA	110 (36)	5 (2)	**<0.0001**
Sm	57 (19)	1 (0.3)	**<0.0001**
RNP 68	28 (9)	0 (0)	**<0.0001**
SSA-Ro52	86 (28)	3 (1)	**<0.0001**
SSA-Ro60	129 (42)	5 (2)	**<0.0001**
SSB	69 (22)	10 (3)	**<0.0001**
Platelet characteristics			
Platelet count (10^9^/l)	235 (188–287)	260 (223–298)	**<0.0001**
Platelet C4d^a^	1.6 (1.3–1.6)	1.2 (1.1–1.3)	**<0.0001**
Platelet size (FSC)	31 900 (29 500–34 200)	33 480 (31 500–25 534)	**<0.0001**
Platelet granularity (SSC)	4255 (3935–4672)	4269 (3980–4269)	0.86
Complement proteins			
Complement factor (C) 3 (g/l)	0.88 (0.70–1.04)	1.05 (0.92–1.20)	**<0.0001**
C4 (g/l)	0.15 (0.10–0.20)	0.21 (0.17–0.25)	**<0.0001**
C3dg (mg/l) (measured in 290 SLE patients)	8.1 (6.2–10.5)	–	
sC5b-9^a^	56 (37–92)	30 (21–44)	**<0.0001**
Other laboratory measurements			
Haemoglobin (g/l)	131 (121–140)	135 (129–141)	**<0.0001**
Leucocyte count (10^9^/l)	4.9 (3.6–6.4)	5.6 (4.7–6.7)	**0.0005**
High-sensitivity CRP^a^ (g/l)	1.4 (0.7–4.7)	1.0 (0.5–2.1)	**<0.0001**
Creatinine (μmol/l)	70 (60–84)	66 (59–7 3)	**<0.0001**
GFR (Cystatin C) (ml/min)	76 (58–101)	108 (93–121)	**<0.0001**
GFR MDRD (ml/min)	86 (68–103)	91 (82–105)	**<0.0001**
sVCAM-1^a^ (ng/l) (12 patients, 6 controls missing)	3924 (315–511)	364 (295–438)	**<0.0001**
IL-6^a^ (ng/l) (87 patients missing)	3.3 (2.2–5.9)	–	ND
IP-10 (pg/l) (8 patients, 8 controls missing)	199 (123–377)	72 (48–100)	**<0.0001**

a Not normally distributed values. IQR: interquartile range; SDI: Lupus International Collaborative Clinics/ACR Damage Index; aβ_2_GPI: anti-β_2_glycoprotein-I; APS: APS according to Miyakis/Sydney criteria; Sm: Smith; FSC: forward scatter; SSC: side scatter; sC5b-9 : soluble complement factor C5b-9; GFR: glomerular filtration rate; MDRD: Modification of Diet in Renal Disease; sVCAM-1: soluble vascular cell adhesion molecule 1; NS: not significant; ND: not determined. Significant *P*-values (<0.05) are given in bold.

### SLE patients have elevated levels of C4d on platelets

**Fig. 1:**
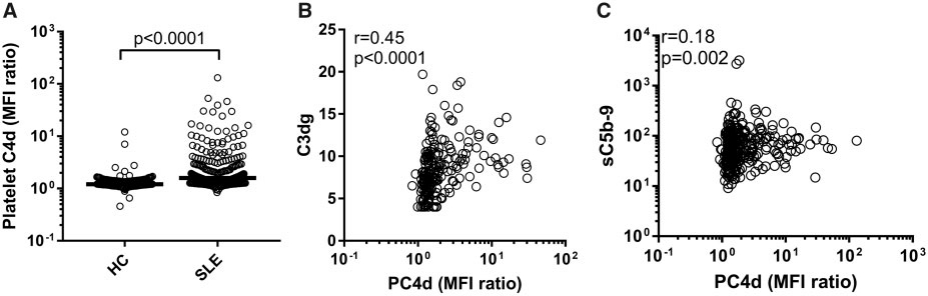
Platelet C4d deposition in population controls and SLE patients (**A**) Platelets were analysed for complement C4d deposition in population controls (*n* = 307) and SLE patients (*n* = 308). (**B** and **C**) Correlation analyses in SLE patients between platelet C4d (PC4d) and (B) complement C3dg activation split product, and (C) terminal complement complex (TCC).

We found more PC4d depositions in SLE patients as compared with population controls (*P* < 0.0001; Table 1, Fig. 1A). PC4d correlated weakly with markers of platelet activation, including platelet CD69 levels (*r*_s_ = 0.12, *P* = 0.04). Further, PC4d correlated with C3dg, a circulating marker of complement activation (measured in 209 of the SLE patients, *r*_s_ = 0.46, *P* < 0.0001; Fig. 1B), and negatively with levels of C3 and C4 (*r*_s_ = –0.28, *P* < 0.0001, and *r*_s_ = –0.35, *P* < 0.0001, respectively). There was also a weak correlation with activation of the terminal complement pathway, measured by sC5B-9 (*r*_s_ = 0.18, *P* = 0.002; Fig. 1C). Consistently, patients with complement consumption, defined according to SLEDAI [34], had elevated PC4d depositions (*P* = 0.007). Using the 95th percentile of the population controls as the cut-off for positivity, 50.3% of the SLE patients were positive for C4d deposition on platelets. Similar to continuous measurements, PC4d positivity was associated with markers of complement activation, and with aPL of all investigated isotypes, LA positivity and with commonly reported high-risk aPL profiles, such as triple positivity [39] and persistent aPL positivity [9]. Additionally, dsDNA antibodies associated positively, and SSB antibodies negatively with PC4d. PC4d was also associated with pro-inflammatory markers such as hsCRP, IP-10, IL-6 and VCAM-1, and weakly with prednisolone treatment, but not with traditional cardiovascular disease risk factors, e.g. age, gender, current smoking and hypertension (Table 2).

**Table 2:** Associations between C4d positivity and characteristics of SLE patients

	PC4d– (*N* = 153)	PC4d+ (*N* = 155)	*P*-value
Demographics and traditional risk factors			
Age (years)	32.4 (14.4)	31.6 (12.9)	0.84
Female sex, %	90.9	93.6	0.38
Current smoking, %	18.3	18.7	0.92
Hypertension, %	42.5	44.8	0.68
Lupus manifestations and characteristics			
Disease duration (years)	15.1 (11.9)	15.1 (11.8)	0.99
Malar rash, %	57.5	50.0	0.25
Photosensitivity, %	73.2	64.5	0.10
Discoid lesions, %	21.0	18.1	0.51
Oral ulcers, %	36.2	35.2	0.72
Arthritis, %	85.6	81.2	0.31
Serositis, %	45.1	32.9	**0.03**
Nephritis, %	37.9	44.5	0.24
CNS manifestation, %	9.1	14.2	0.17
Leucopenia, %	50.7	50.7	1.00
Thrombocytopenia, %	16.6	25.3	0.06
Immunologic criteria	57.5	81.3	**<0.0001**
SLICC damage index >1%	65.4	62.6	0.61
SLAM >6, %	60.1	60.6	0.93
SLEDAI >6	26.1	38.7	**0.02**
Treatment (at inclusion)			
Steroids, %	50.3	61.9	**0.04**
Antimalaria agents, %	32.7	31.8	0.87
aPL/disorders (positivity %)			
aCL IgG	10.5	38.7	**<0.0001**
aCL IgM	1.3	9.0	**0.002**
aCL IgA	7.2	25.2	**<0.0001**
aβ_2_GPI IgG	10.5	41.3	**<0.0001**
aβ_2_GPI IgM	3.3	11.0	**0.009**
aβ_2_GPI IgA	5.9	25.2	**<0.0001**
LA	5.3	27.1	**<0.0001**
Any aPL	15.1	47.1	**<0.0001**
Persistent aPL	6.0	36.8	**<0.0001**
Triple aPL positivity	2.6	24.5	**<0.0001**
APS, %	0.7	18.1	**<0.0001**
Other autoantibodies, %			
ANA (ever)	98.7	98.7	1.0
dsDNA (ever)	54.0	80.9	**<0.0001**
dsDNA (at inclusion)	28.8	42.6	**0.01**
Sm	15.0	21.9	0.12
RNP 68	7.8	10.3	0.45
SSA-Ro52	31.4	24.5	0.18
SSA-Ro60	45.8	38.1	0.17
SSB	30.7	14.2	**0.0005**
Platelet characteristics			
Platelet count (10^9^/l)	232 (198–282)	235 (173–296)	0.93
Platelet size (FSC)	31 688 (34 120–29 344)	32 161 (34 472–29 917)	0.51
Platelet granularity (SSC)	4223 (3927–4588)	4276 (3942–4747)	0.35
Complement proteins			
Complement factor (C) 3 (g/l)	0.95 (0.75–1.09)	0.8 (0.66–0.97)	**<0.0001**
C4 (g/l)	0.17 (0.12–0.22)	0.13 (0.08–0.17)	**<0.0001**
C3dg (mg/l) (measured in 211 SLE patients)	7.2 (5.7–9.4)	9 (7.4–11.0)	**<0.0001**
sC5b-9^a^ (4 missing)	50.5 (33.0–81.8)	67.8 (40.2–104.8)	**0.003**
Other laboratory measurements			
Haemoglobin (g/l)	130 (118–140)	132 (122–141)	0.10
Leucocyte count 10^9^/l	4.9 (3.5–7.0)	4.9 (3.6–6.2)	0.63
High-sensitivity CRP^a^ (mg/l)	1.1 (0.5–3.4)	1.9 (0.8–6.1)	**0.0002**
Creatinine (μmol/l)	71 (60–85)	69 (60–83)	0.10
GFR (Cystatin C) (ml/min)	76 (58–104)	76 (57–99)	0.91
GFR MDRD (ml/min)	85 (67–1 01)	88 (68–103)	0.54
sVCAM-1^a^ (ng/l)	387 (300–4876)	410 (327–530)	**0.02**
IL-6^a^ ng/l (87 missing)	3.0 (2.2–5.2)	4.1 (2.4–7.6)	**0.02**
IP-10 pg/l (8 missing)	174 (111–281)	236 (140–425)	**0.002**

a Not normally distributed values. Data are presented as mean (S.d.) or median (interquartile range) unless otherwise stated. PC4d: platelet C4d; SDI: Lupus International Collaborative Clinics/ACR Damage Index; aβ_2_GPI: anti-β_2_glycoprotein-I; APS: APS according to Miyakis/Sydney criteria; Sm: Smith; FSC: forward scatter; SSC: side scatter; sC5b-9:  soluble complement factor C5b-9; GFR: glomerular filtration rate. MDRD: Modification of Diet in Renal Disease; sVCAM: soluble vascular cell adhesion molecule. Significant *P*-values (<0.05) are given in bold.

### PC4d and aPL are associated with a history of vascular events in patients with SLE

Previous vascular events of any type were increased 3-fold in SLE patients who were positive for PC4d. This positive association was primarily driven by VTE and ischaemic stroke, while IHD did not contribute. PC4d was not associated with subclinical atherosclerosis, as measured by carotid plaques occurrence (Table 3).

**Table 3:** Associations between PC4d positivity (+) and LA+ with vascular manifestations among SLE patients

Clinical manifestation/characteristic (numbers: yes/no)	PC4d+ [OR (95% CI)]	*P*-value, PC4d	LA+ [OR (95% CI)]	*P*-value, LA
Any vascular event (82/226)	2.9 (1.7, 4.9)	<0.0001	6.2 (3.3, 12.0)	<0.0001
Any arterial event (45/263)	2.0 (1.1, 3.8)	**0.03**	2.9 (1.4, 5.8)	**0.002**
Ischaemic heart disease (18/219)	1.3 (0.5, 3.2)	0.51	1.2 (0.4, 3.8)	0.72
Myocardial infarction (15/293)	0.7 (0.2, 1.9)	0.44	0.8 (0.2, 2.8)	0.69
Ischaemic cerebrovascular disease (30/278)	1.8 (0.8, 3.9)	0.14	4.2 (1.9, 9.4)	**0.0002**
Ischaemic stroke (24/284)	2.6 (1.03, 6.4)	**0.04**	5.3 (2.2, 12.7)	<0.0001
Carotid plaque (57/224)	0.9 (0.5, 1.6)	0.68	1.4 (0.6, 3.3)	0.92
Any venous thromboembolism (46/262)	2.9 (1.5, 5.8)	**0.002**	5.2 (2.6, 10.5)	<0.0001
APS (29/276)	32.8 (4.4, 244.8)	<0.0001	LA part of definition	–
LA (50/307)	6.7 (3.0, 14.8)	<0.0001	N/A	N/A

PC4d: platelet C4d; OR: odds ratio; N/A: not applicable. Significant *P*-values (<0.05) are given in bold.

Further, we tested all the investigated variables for associations with any vascular event. Of the traditional risk factors, age and hypertension, and among lupus-associated risk factors, all aPL, except those of IgM isotype, complement degradation products PC4d and C3dg, measures of renal involvement (nephritis ever) and impaired renal function, sVCAM-1 and prednisolone treatment, were all associated with vascular events. Negative associations were observed for serositis and SSB antibodies (supplementary Table S1, available at *Rheumatology* online).

High-risk aPL profiles were specifically tested for associations with specific vascular events, and we could confirm previous reports that the strongest association with vascular events was observed for LA positivity [40] (supplementary Table S2, available at *Rheumatology* online). Therefore, we used LA positivity as a proxy for aPL in further analyses. Associations between LA and history of vascular events/measures are presented in Table 3, demonstrating positive associations with all investigated types of events, except IHD and myocardial infarction. LA was not associated with carotid plaques.

### Platelet C4d has added clinical value in identifying patients with vascular events

Due to the limited number of events we were only powered to perform multivariable analyses for the combined vascular outcomes. In these models, PC4d positivity remained associated with any vascular event (OR: 2.4, 95% CI: 1.3, 4.6, *P* = 0.006) after adjustment for age, gender, hypertension, smoking, glomerular filtration rate according to the Modification of Diet in Renal Disease, steroid treatment and LA. Any VTE was also associated with PC4d, but with borderline significance (OR: 2.1; 95% CI: 1.0, 4.4, *P* = 0.050), after adjustment for age, gender and LA. Arterial events were not associated with PC4d positivity after adjustment for age, gender, hypertension and LA. The full multivariable models are presented in supplementary Table S3A–C, available at *Rheumatology* online.

**Fig. 2:**
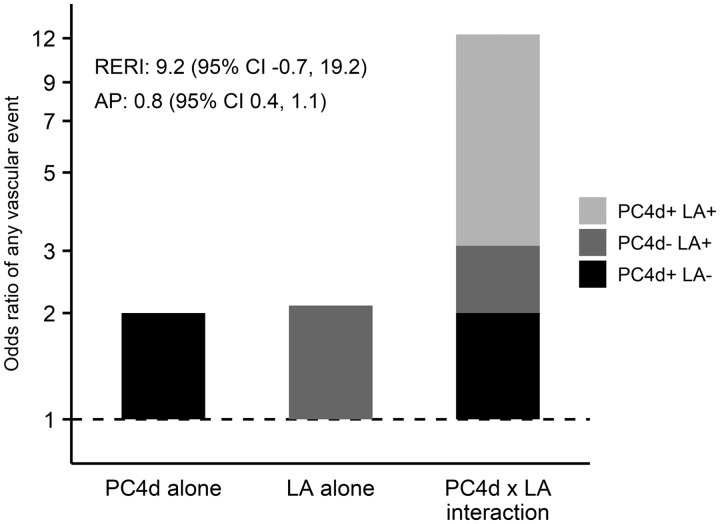
Interaction between PC4d depositions and lupus anticoagulant on the odds of any previous vascular disease. Interaction between platelet complement C4d deposition (PC4d) and LA, after adjustment for age (in 10 years), sex, hypertension, MDRD (per 10 units), smoking and steroid treatment on the odds of vascular disease in individuals with SLE. RERI: relative excess risk due to interaction; AP: attributable proportion due to interaction; MDRD: Modification of Diet in Renal Disease. See supplementary Table S4, available at *Rheumatology* online for further information.

To further investigate if PC4d contributes to vascular events independently of aPL, we stratified SLE patients for the four possible combinations of PC4d and LA positivity/negativity. SLE patients who were negative for both PC4d and LA were used as reference. Our results demonstrate a positive interaction between PC4d and LA positivity for a history of any vascular event, with an attributable proportion of 0.8 (95% CI: 0.4, 1.1). The relative excess risk was 9.2, though with a CI overlapping 0, possibly indicating lack of power (95% CI: −0.7, 19.2). These results are presented in Fig. 2 and the full calculations can be found in supplementary Table S4, available at *Rheumatology* online.

## Discussion

In this large study, comprising >300 SLE patients and 300 representative controls, we demonstrate that depositions of a complement degradation product, C4d, on platelets is frequent in SLE, but is scarce in controls. PC4d was an independent marker of previous vascular events in SLE, and when combined with LA, it added additional information to the assessment of vascular risk in patients with SLE.

We recently observed that aPL antibodies fix complement on platelets, promoting a procoagulant phenotype [17]. We have also reported that SLE patients had increased PC4d depositions, and that these were in particular present in patients with cardiovascular disease [17]. Though clearly associated with vascular disease, it is not known whether PC4d adds to the current panel of vascular risk markers in SLE, e.g. traditional cardiovascular risk factors, aPL antibodies and nephritis/impaired renal function [10–12].

Platelet activation has been repeatedly reported in SLE [18–20], and it has been associated with the increased cardiovascular morbidity and mortality observed in these patients [7, 10]. Several platelet activation triggers have also been identified, including immune complexes, shear stress, damaged endothelium, oxidized low-density lipoprotein, inflammatory mediators and aPL antibodies [18, 41, 42], likely operating jointly to amplify platelet activation and promote thrombosis. aPL antibodies have in this setting been suggested to act as a second hit, further enhancing platelet activation, once primed with primary stimuli [17]. When activated, platelets expose several molecules, including phosphatidylserine, P-selectin and chondroitin sulfate, all of which can initiate the classical pathway of the complement system [21, 43]. Anti-platelet antibodies, as well as aPL, may also directly serve as initiators of the classical pathway when bound to the platelet surface [17]. All these observations are consistent with the current findings of PC4d deposition being more prevalent in the presence of aPL antibodies.

What then are the consequences of complement activation on platelets? Though clearly associated with severe outcomes, such as cardiovascular disease and venous thrombosis [17, 25, 27], the underlying mechanisms contributing to these events are not fully understood. Platelets express several complement receptors, such as gC1qR, cC1qR, C3aR and C5aR, all of which have been implicated as promotors of platelet activation and aggregation [44, 45]. When complement activation proceeds to formation of the terminal membrane attack complex, platelets will lyse and form thrombin-generating microparticles [24] and the pro-inflammatory split products C3a and C5a. Though we did not study the underlying mechanisms of microparticle formation, we recently reported that platelet-derived microparticles are up to 10 times more abundant in SLE patients than in controls [46]. Complement activation may also act indirectly on the endothelium, to support a pro-thrombotic environment [47]. Consistent with this interpretation, we found a positive correlation between PC4d levels and VCAM-1. PC4d associated better with any vascular disease than the other investigated complement proteins in this study, but we also observed a positive association with C3dg (supplementary Table S1, available at *Rheumatology* online), a degradation product of C3 (measured in a subset of 211 patients), indicating that PC4d is not the only complement measure associated with vascular disease. Though *in vivo* models have demonstrated a clear role of complement in cardiovascular disease [47–49], further studies are needed to understand which particular receptors and cells are involved in mediating the pro-thrombotic effects of complement.

As clearly demonstrated in the current investigation, and in prior studies, PC4d depositions are associated with vascular disease in SLE [17, 25, 27–29]. Petri *et al.* recently suggested that a composite risk score, including PC4d, LA and low C3, was associated with recent thrombosis in SLE [50]. We here demonstrate that two of these risk factors, C4d and LA, are independently and synergistically associated with vascular events. However, we cannot confirm positive associations with low C3 levels. Though initially suggested to be selective and diagnostic for SLE [26], current literature support the presence of PC4d also in RA [17] and SSc [17], and in stroke patients from the general populations [27], e.g. other conditions with increased platelet activation and cardiovascular morbidity.

PC4d has previously been associated with disease activity in SLE patients [26]. In our study, including >300 SLE patients, we were unable to find any association with disease activity, measured by both SLEDAI-2K and SLAM indices. This is consistent with data from a prior study on 150 SLE patients [17]. There was, however, a positive association between PC4d and dsDNA antibodies, which together with positive associations with measures of complement activation may explain the previously observed association to disease activity, estimated by SLEDAI-2K, where both these laboratory items are included. Age, gender, disease duration and organ damage (Lupus International Collaborative Clinics/ACR Damage Index) were not associated with PC4d. However, we did observe higher levels of hsCRP and pro-inflammatory cytokines IP-10, IL-6 and endothelial marker VCAM-1, often associated with systemic inflammation, among patients who were positive for PC4d. We also noted that PC4d was less frequent among SSB-positive SLE patients, an SLE subgroup reported to be at reduced risk of cardiovascular disease [11]. Apart from vascular disease, PC4d was not associated with any particular SLE manifestation.

Though clearly associated with complement activation and consumption, it should be noted that the antibody clone used to detect PC4d recognizes all C4d-containing complement components, including naïve C4. While platelets can deposit naïve complement components on their cell surface [21], prior work from our group demonstrates that complement components observed on the platelet surface in SLE patients are proteolytically cleaved, including C4 [25]. Future studies should be conducted with antibodies recognizing the cleaved C4d neo epitope to firmly establish whether PC4 or PC4d is related to vascular disease in SLE.

Arterial thromboses/occlusions are commonly used as a composite outcome including IHD, ICVD and ischaemic peripheral arterial disease. Rupture of atherosclerotic plaques are often assumed to be the cause of these events, but emboli from more centrally situated thrombotic lesions are also common. Emboli are of thrombotic origin and a more frequent cause of ICVD than of IHD. According to our observations PC4d was associated with ICVD and VTE, but not with IHD or atherosclerosis as measured by carotid plaques, suggesting that PC4d is primarily associated with a high risk of thromboembolic disease.

The current study builds on previous investigations demonstrating elevated PC4d in SLE in association with vascular disease. In the largest SLE cohort so far analysed for PC4d, we were able to validate prior findings that PC4d is associated with vascular events using frozen platelets. Though this represents a technical advance, the novelty of the present study is the comparison and positive interaction between PC4d and LA. LA, as well as aPL antibodies, are widely used in the clinical setting to diagnose the APS and to assess the risk of thrombosis. Of the aPL, LA is the strongest predictor of vascular event [40]. Despite low numbers, our findings suggest that dual PC4d and LA may function synergistically in SLE-related vascular disease. PC4d, a proxy for complement activation on platelets, is alone clinically important [29], but even more so when combined with LA. Future prospective studies will validate whether the prognostic capacity of LA can be improved by considering PC4d and LA together.

Overall, our study suggests that PC4d is an independent marker of vascular disease in SLE, which when combined with LA, could add clinical value to the assessment of the risk for vascular events. Future studies are warranted to assess the prognostic capacity of PC4d.

## Supplementary Material

keaa092_supplementary_data
