# Supplementary material for: Complement deposition, C4d, on platelets is associated with vascular events in systemic lupus erythematosus
Source: Rheumatology (Oxford). 2020 Apr 7;59(11):3264–74. doi: 10.1093/rheumatology/keaa092 (PMC7590416; doi:10.1093/rheumatology/keaa092)
Supplement: keaa092_supplementary_data [file keaa092_supplementary_data.zip › rhe-19-1989-File005.docx]

**SUPPLEMENTARY MATERIAL**

**Definition of vascular events**

1) Ischemic cerebrovascular disease: Stroke including cerebral infarction, confirmed by computer tomography or magnetic resonance imaging and/or transient ischemic attacks, defined as focal symptoms from the brain or retina with a maximum duration of 24 hours.

2) Ischemic heart disease: Myocardial infarction, confirmed by electrocardiography and a rise in plasma creatine kinase-MB or troponin T and/or angina pectoris confirmed by exercise stress test.

3) Ischemic peripheral vascular disease: Intermittent claudication and/or peripheral arterial thrombosis or embolus confirmed by angiogram or Doppler flow studies.

4) Any arterial event: Any of 1-3.

5) Venous thromboembolism: Deep vein thrombosis, confirmed by venography or ultrasonography and/or pulmonary embolism, confirmed by radionuclide lung scanning or angiogram.

**Supplementary table S1** **Associations between traditional risk factors, lupus characteristics, autoantibodies and inflammatory markers and “any vascular event”.**

| ***Demographics and traditional risk factors*** | **Any vascular event OR (95% CI)** | **P** |
| --- | --- | --- |
| **Age (/ 10 years)** | **1.43 (1.19 – 1.72)** | **<0.001** |
| **Female sex** | **0.84 (0.54 – 1.31)** | **0.46** |
| **Current smoking** | **1.22 (0.65-2.29)** | **0.47** |
| **Hypertension** | **2.28 (1.36- 3.82)** | **0.0017** |
|  |  |  |
| ***Lupus manifestations and characteristics*** |  |  |
| Disease duration (/10 years) | 1.19 (0.97 – 1.47) | 0.10 |
| Malar rash | 0.95 (0.76 – 1.23) | 0.87 |
| Photosensitivity | 0.98 (0.75 – 1.29) | 0.90 |
| Discoid lesions | 0.90 (0.64 – 1.25) | 0.51 |
| Oral ulcers | 1.02 (0.78 – 1.33) | 0.89 |
| Arthritis | 0.98 (0.70 – 1.37) | 0.88 |
| Serositis | 0.76 (0.59 – 0.98) | 0.034 |
| Nephritis | 1.46 (1.13- 1.89) | 0.0037 |
| CNS manifestation | 0.78 (0.54 – 1.12 | 0.17 |
| Leucopenia |  |  |
| Lymphopenia | 0.89 (0.69 – 1.14) | 0.35 |
| Thrombocytopenia | 0.94 (0.69- 1.14) | 0.35 |
| Immunologic criteria | 0.93(0.54- 1.60) | 0.79 |
| SLICC damage index>1 | ND |  |
| SLAM>6 | 0.98 (0.76 – 1.27) | 0.90 |
| SLEDAI >6 | 0.85 (0.65 – 1.11) | 0.23 |
|  |  |  |
| **Treatment (at inclusion)** |  |  |
| Prednisolone, yes/no | 2.01 (1.18 – 3.42) | 0.010 |
| Anti-malaria medication, yes/no | 0.76 (0.44 – 1.33) | 0.34 |
|  |  |  |
| ***Antiphospholipid antibodies/disorders (positivity %)*** |  |  |
| aCL IgG | 3.10 (1.79 – 5.39) | **<0.001** |
| aCL IgM | 1.27 (0.43 – 3.77) | 0.67 |
| aCL IgA | 2.87 (1.53 – 5.38) | **<0.001** |
| aβ_2_GPI IgG | 3.00 (1.74-5.17) | **<0.001** |
| aβ_2_GPI IgM | 1.04 (0.39 – 2.75) | 0.94 |
| aβ_2_GPI IgA | 2.87 (1.53 – 5.38) | **<0.001** |
| **Lupus anticoagulant** | **6.59 (3.45-12.60)** | **<0.001** |
| Any aPL | 3.58 (2.10 – 6.09) | **<0.001** |
| Persistent aPL | 3.00 (1.69 – 5.32) | **<0.001** |
| Triple aPL positivity | 6.09 (3.06 – 12.14) | 2.7 x 10-7 |
| APS | ND |  |
|  |  |  |
| ***Other autoantibodies (positivity %)*** |  |  |
| ANA (ever) | 1.05 (0.34 -3.27) | 0.94 |
| dsDNA (ever) | 1.07 (0.81 – 1.41) | 0.65 |
| dsDNA (at inclusion) | 1.05 (0.62 - 1.78) | 0.84 |
| Sm | 0.98 (0.51 – 1.88) | 0.95 |
| RNP 68 | 1.11 (0.47 – 2.64) | 0.81 |
| SSA-Ro52 | 0.72 (0.40 – 1.29) | 0.26 |
| SSA-Ro60 | 0.64 (0.38 – 1.09) | 0.10 |
| SSB | 0.39 (0.19 – 0.81) | 0.012 |
|  |  |  |
| ***Platelet characteristics*** |  |  |
| Platelet count/ 25 x 10^9^/L | 1.06 (0.83 – 1.37) | 0.64 |
| Platelet size (FSC)/SD | 0.96 (0.74 – 1.23) | 0.73 |
| Platelet granularity (SSC) | 1.00 (0.99 -1.02) | 0.78 |
|  |  |  |
| ***Complement proteins*** |  |  |
| Complement factor (C) 3 g/L | 1.03 (0.80 – 1.33) | 0.79 |
| C4 g/L | 0.99 (0.77 – 1.28) | 0.96 |
| C3dg mg/L (measured in 211 SLE patients) | 1.51 (1.11 – 2.06) | 0.0084 |
| sC5b-9* (4 missing) | 1.08 (0.83 – 1.39) | 0.58 |
| **PC4d 95% cut-off #** | **2.98 (1.74 – 5.10)** | **<0.001** |
|  |  |  |
| ***Other laboratory measurements*** |  |  |
| Hemoglobin g/L | 0.90 (0.70 – 1.17) | 0.44 |
| Leucocyte count 10^9^/L | 1.20 (0.94 – 1.53) | 0.15 |
| High-sensitivity CRP* mg/L | 1.19 (0.92 – 1.54) | 0.18 |
| Creatinine μmol/L | 1.34 (1.05 – 1.70) | 0.019 |
| GFR (Cystatin C) ml/min | 0.72 (0.55 – 0.93) | 0.013 |
| **GFR MDRD ml/min #** | **0.65 0.50 – 0.84)** | **0.0013** |
| sVCAM-1* ng/L | 1.31 (1.01 – 1.69) | 0.040 |
| IL-6 ng/L (87 missing) | 0.87 (0.63 – 1.20) | 0.39 |
| IP-10 pg/L (8 missing) | 0.98 (0.76 – 1.27) | 0.90 |

CNS: Central nervous system; SDI: Lupus International Collaborative Clinics/American College of Rheumatology Damage Index; SLAM: SLE activity measure; SLEDAI: SLE Disease Activity Index; aCL, Ig: Immunoglobulin; aβ_2_GPI: anti-β_2_glycoprotein-I; aPL: antiphospholipid antibodies; APS: Antiphospholipid syndrome according to Miyakis/Sydney criteria; ANA: anti-nuclear antibodies; dsDNA: double stranded DNA; Sm: Smith; RNP: ribonucleoprotein; SSA/SSB Sjögren’s syndrome antigen A/B, sC5b-9: soluble complement factor C5b-9; GFR: glomerular filtration rate; MDRD: Modification of Diet in renal Disease; sVCAM: soluble vascular cell adhesion molecule; IL: interleukin. *Not normally distributed values, analyzed after log transformation. # Selected for inclusion in the multivariable analyses.

**Supplementary Table S2 Associations between “high risk aPL profiles” and specific vascular events among patients with SLE**

|  | **LA+** | **Any aPL+** | **Triple aPL +** |
| --- | --- | --- | --- |
| Ischemic heart disease % | 1.2 (0.4-3.8) | 1.1 (0.4-2.8) | 1.0 (0.3-3.8) |
| Myocardial infarction % | 0.8 (0.2-2.8) | 1.2 (0.4-4.1) | 0.6 (0.2-2.3) |
| Ischemic cerebrovascular disease % | **4.2 (1.9-9.4)** | **2.4 (1.1-5.1)** | **2.6 1.1-6.3)** |
| Ischemic stroke | **5.3 (2.2-12.6)** | **3.4 (1.5-8.0)** | **2.9 (1.1-7.6)** |
| Any arterial event % | **2.9 (1.4-5.9)** | **2.1 (1.1-3.9)** | **2.2 (1.0-4.7)** |
| Venous thromboembolism % | **5.2 (2.6- 10.5)** | **3.2 (1.7-6.0)** | **6.4 (3.1-13.2)** |
| Any vascular event (arterial or venous) % | **6.3 (3.3-12.0)** | **3.4 (2.0-5.7)** | **5.8 (2.9-11.6)** |

Associations are presented as odds ratios and 95% confident intervals

**Supplementary Table S3 Multivariable logistic regression models for vascular outcomes and PC4d in patients with SLE**

1. Multivariable analysis for any vascular event (yes=82, no=226)

|  | OR | 95% CI | P-value |
| --- | --- | --- | --- |
| **Age/10 years** | **1.5** | **1.2-1.8** | **0.001** |
| Gender, female | 0.9 | 0.5-1.5 | 0.60 |
| Hypertension | 1.4 | 0.8-2.6 | 0.25 |
| Smoking | 1.2 | 0.6-2.5 | 0.48 |
| MDRD/10 | 1.0 | 0.9-1.1 | 0.69 |
| **Steroid treatment** | **1.9** | **1.0-3.6** | **0.04** |
| **LA** | **4.9** | **2.4-9.9** | **<0.0001** |
| **PC4d (positivity)** | **2.3** | **1.3-4.3** | **0.008** |

1. Multivariable analysis for any arterial event (yes=45, no=263)

|  | OR | 95% CI | P-value |
| --- | --- | --- | --- |
| **Age/10 years** | **1.9** | **1.4-2.5** | **<0.0001** |
| Gender female | 1.5 | 0.7-3.4 | 0.28 |
| Hypertension | 1.9 | 0.9-4.0 | 0.08 |
| **LA** | **2.5** | **1.1-5.6** | **0.03** |
| PC4d | 1.8 | 0.9-4.0 | 0.10 |

1. Multivariable analysis for any venous thromboembolism (yes=46, no=262)

|  | OR | 95% CI | P-value |
| --- | --- | --- | --- |
| Age/10 years | 1.0 | 0.8-1.3 | 0.89 |
| Gender female | 0.6 | 0.4-1.0 | 0.051 |
| **LA** | **4.0** | **1.9-8.4** | **0.0003** |
| **PC4d** | **2.1** | **1.0-4.4** | **0.050** |

LA: lupus anticoagulant; PC4d: Platelet C4d deposition. + =positive if > 95^th^ percentile of the population controls, otherwise negative= -, OR: odds ratio; CI: confidence interval

**Supplementary Table S4 Interaction analysis for any vascular event**

|  | **OR (95% CI)** | | |
| --- | --- | --- | --- |
|  | **PC4d-** | **PC4d+** | |
| **LA-** | 1.0 [Referent] | 2.0 (1.0-3.9) | |
| **LA+** | 2.1 (0.4-9.6) | 12.3 (5.4-29.3) | |
|  |  |  | |
| ***Multiplicative interaction,***  ***ratio of ORs (95% CI)*** | | | 3.0 (0.5-19.8) |
| ***Additive interaction, RERI (95% CI)*** | | | 9.2 (-0.7-19.2) |
| ***Additive interaction, AP (95% CI)*** | | | 0.8 (0.4-1.1) |

OR: odds ratio; CI: confidence interval; LA: lupus anticoagulant; PC4d: Platelet C4d deposition; RERI: relative excess risk due to interaction; AP: attributable proportion due to interaction.

PC4d considered positive if >95^th^ percentile of the population controls’ level, otherwise negative. Odds ratios estimated from logistic regression model adjusted for age (in 10 years), gender, hypertension, smoking, and MDRD (per 10 mL/min/1.73 m²), and prednisolone treatment (yes/no).

RERI can be calculated using the equation: $\text{RERI}={OR}_{\text{PCd4+LA+}}-{OR}_{\text{PC4d+LA-}}-{OR}_{\text{PC4d-LA+}}+1$ and AP using the equation: $\text{AP}=\frac{\text{RERI}}{{OR}_{\text{PC4d+LA+}}}$.

**Supplementary figure legend**

**Supplemental Figure S1. Frozen platelet analysis.** Platelet-rich plasma (RPP), either obtained immediately or frozen (fPRP) were analyzed for complement C4d deposition on platelets using flow cytometry. In total, PRP from nine individuals were analyzed. Statistics were analyzed using Spearman’s correlation test.
