# Supplementary figures and images for: Complement deposition, C4d, on platelets is associated with vascular events in systemic lupus erythematosus
Source: Rheumatology (Oxford). 2020 Apr 7;59(11):3264–74. doi: 10.1093/rheumatology/keaa092 (PMC7590416; doi:10.1093/rheumatology/keaa092)

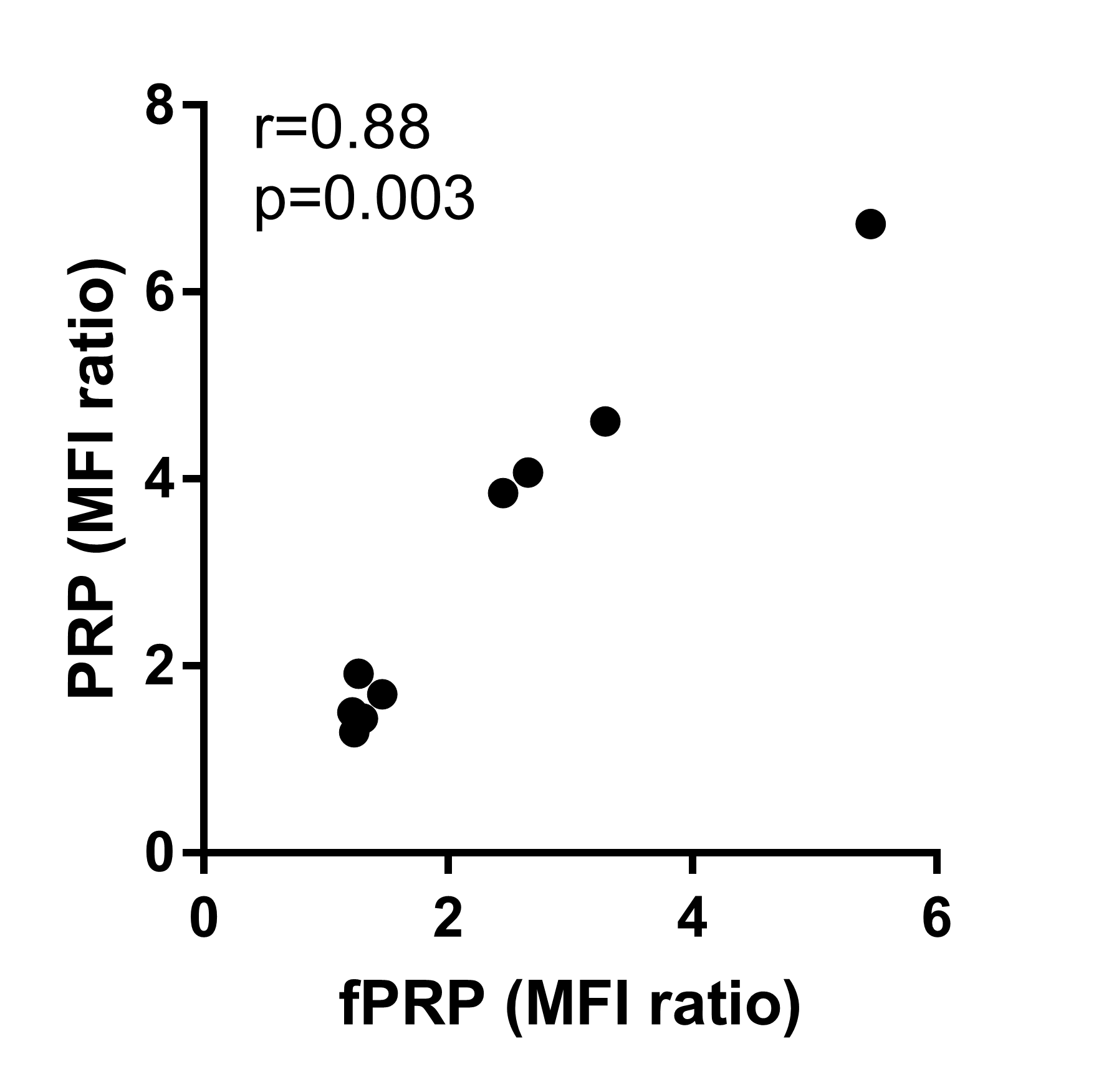

Supplement: keaa092_supplementary_data [file keaa092_supplementary_data.zip › rhe-19-1989-File006.tif]
